# 

**Published:** 1959-04

**Authors:** 


MEDICAL JOURNAL OF THE SOUTH-WEST

*(Incorporating the Bristol Medico-Chirurgical Journal)*

Established 1883

VOL. 74 (ii) | April 1959 | NO. 272

### Contents

| | | Page |
|---|---|---|
| A REVIEW OF ACUTE INTESTINAL OBSTRUCTION | *Mr. P. J. W. Monks* | 31 |
| SQUINT . . . . . . | *Dr. Calbert I. Phillips* | 40 |
| TRANSPORTING SEVERELY PARALYSED PATIENTS | *Dr. James Macrae* | 45 |
| APLASTIC ANAEMIA WITH POST-TRANSFUSIONAL HAEMOSIDEROSIS | *Clinical Pathological Conference* | 47 |
| NOTES AND NEWS . . . . . . . | | 51 |
| APPOINTMENTS . . . . . . . . | | 51 |

*PUBLISHED QUARTERLY*

*ANNUAL SUBSCRIPTION 16/- POST FREE*

PUBLISHED UNDER THE AUSPICES OF THE

BRISTOL MEDICO-CHIRURGICAL SOCIETY

### *Editorial Committee*

Dr. J. E. Cates, Department of Medicine, Bristol Royal Infirmary. *Editor.*

Dr. N. J. Brown, Southmead Hospital, Bristol. *Assistant Editor.*

Dr. J. Macrae, Ham Green Hospital.

Dr. R. C. Wofinden, Central Clinic, Bristol.

Mr. A. E. S. Roberts, Medical Library, University of Bristol. *Secretary.*

### *Local Correspondents*

*Bath* . . Mr. A. D. Bateman, 3 The Circus, Bath.

*Exeter* . . Dr. L. N. Jackson, Mount Jocelyn, Crediton, Devon.

*Gloucester* . Dr. R. F. Jarrett, 9 College Green, Gloucester.

*Plymouth* . Dr. C. R. Croft, Lexden, Hartley Av., Plymouth.

*Taunton* . Dr. M. McAlley, 1 The Crescent, Taunton.

*Torquay* . Dr. A. Robinson Thomas, 20 Devon Square, Newton Abbot, Devon.

*Truro* . . Dr. F. D. M. Hocking, St. Andrews, Perranporth, Cornwall.

*Yeovil* . . Mr. J. A. Vere Nicoll, Knapp House, Yeovil, Somerset.

### NOTICE TO CONTRIBUTORS

Original articles are invited, provided they have not been published elsewhere. Notes of forthcoming events, meetings held, degrees conferred, staff appointments, and other local news are welcomed. The editorial committee reserves the right to make literary corrections.

Illustrations should always be supplied ready for reproduction. All re-touchings or other operations required will be charged to the author. Line drawings should be drawn in Indian ink. We regret that we must ask authors to pay for making blocks, if this is necessary.

J. W. ARROWSMITH LTD., WINTERSTOKE ROAD,

BRISTOL.

### Notes and News

New Year Honour: G. C. Kelly, C.B.E. (Civil Division).

Professor J. M. Yoffey has been awarded the order of "Knight of Dannebrog" for his services to Danish medical students.

Professor G. Gordon Lennon has been elected an honorary member of the Turkish Society of Gynaecology.

The Spring Dinner of the Wessex Rahere Club will be held in the Royal Clarence Hotel, Exeter, on Saturday, April 18th. Further details may be had from Mr. A. Daunt Bateman, 11 The Circus, Bath.

EXAMINATION RESULTS

*UNIVERSITY OF BRISTOL*

*M.D.*: P. C. Farrant, K. R. Gough, M. D. Turner.

*Ph.D.*: H. S. M. Crabb, D. G. Lyon.

### Appointments

F. G. Bolton, M.D., M.R.C.P., Consultant Clinical Pathologist, Churchill Hospital, Oxford.

A. G. Freeman, M.A., M.D., M.R.C.P., Consultant Physician, Swindon and Cirencester area.

Joyce Fung, M.B., M.O. British Guiana.

Brenda Grant, M.B. (Liverpool), D.P.W., Supervising Psychiatrist, Gracie Square Hospital, New York.

*UNITED BRISTOL HOSPITALS*

*DECEMBER*, 1958 *TO FEBRUARY* 1959

| *Name* | *Appointment* | *Formerly* |
|---|---|---|
| Evans, K. T., M.B., CH.B., M.R.C.P., D.M.R.D., F.F.R. | Consultant Radiologist. | Senior Registrar in Radiodiagnosis, Hammersmith Hospital & Postgraduate Medical School. |
| Barritt, D. W., M.D., M.R.C.P. | Assistant Cardiological Physician. | Lecturer in Medicine, University of Bristol (Senior Registrar status). |

*SOUTH WESTERN REGIONAL HOSPITAL BOARD*

*DECEMBER*, 1958 *TO FEBRUARY*, 1959

*BRISTOL*

| *Name* | *Appointment* | *Formerly* |
|---|---|---|
| Johnson, D. H., M.D. (Bristol) | Consultant Pathologist, Weston-super-Mare General Hospital. | Lecturer in Pathology, University of Bristol. |
| Poley, B. A., M.B., CH.B. (Bristol), D.A. | Anasthetice Registrar, Southmead Hospital. | S.H.O. Anaesthetist, United Bristol Hospitals. |
| White, Miss C. M., MB., CH.B. (Glasgow), D.C.H. (London). | Paediatric Registrar, Southmead Hospital. | Paediatric Registrar, Nottingham Children's Hospital. |
| Mitra, A. K., M.B., B.S. (Calcutta). | Registrar to Dept. of Pathology Frenchay Hospital. | S.H.O. in Pathology, Bolton District General Hospital. |
| Roylance, J., M.B., CH.B. (Bristol), D.M.R.D. | Registrar in Radiodiagnosis, Southmead Hospital. | Short service commission in R.A.M.C. Junior Specialist in Radiology. |

| *Name* | *Appointment* | *Formerly* |
|---|---|---|
| WEBB, A. J., M.B., CH.B. (Bristol). | Surgical Registrar, Cossham-Frenchay Group of Hospitals. | S.H.O. in General Surgery, Frenchay Hospital. |

### *NORTH GLOUCESTERSHIRE*

| *Name* | *Appointment* | *Formerly* |
|---|---|---|
| CHIPPINDALE, F. W., N.B., B.S. (London), D.A. (England), F.F.A.R.C.S. | G.P. Anaesthetist, Dilke Memorial Hospital, Cinderford. | Royal Navy — Surgeon Commander. |
| WARD, F. W., M.B., B.S. (London). | Surgical Registrar, Cheltenham General Hospital. | Casualty Officer & Radiotherapy House Surgeon, Essex County Hospital. |
| CARDALE, J. D., M.B., CH.B. (Bristol). | G.P. Surgeon, Lydney & District Hospital. | In general practice in Lydney. |

### *BATH*

| *Name* | *Appointment* | *Formerly* |
|---|---|---|
| KELLY, T. STUART-BLACK, M.B., CH.B. (Manchester), F.R.C.S.ED., D.O.M.S. | Consultant Ophthalmic Surgeon, Bath Eye Infirmary. | Consultant Ophthalmic Surgeon, Victoria Eye Hospital, Hereford. |
| WINTERTON, A. J., M.B., B.S. (Lond.), D.R.C.O.G., D.C.H. | G.P. Anaesthetist, Chippenham Hospital. | In general practice in Chippenham. |
| BEARD, Miss M. F., B.A., M.B., B.CHIR. (Cantab.) | Anaesthetic Registrar, Bath Group of Hospitals. | S.H.O. to Anaesthetic Dept. United Norwich Hospitals. |

### *DEVON AND CORNWALL*

| *Name* | *Appointment* | *Formerly* |
|---|---|---|
| MONKS, P. J. W., M.B., B.S. (Lond.), F.R.C.S. (Eng.). | Consultant Surgeon, Devon and Exeter Clinical Area (Torquay). | Senior Surgical Registrar, United Bristol Hospitals. |
| PENCHEON, J. M., M.B., CH.B. (Leeds), D.P.M. | Consultant Psychiatrist, West Cornwall Clinical Area. | Assistant Psychiatrist, Lancaster Moor Hospital, Lancaster. |
| RODGER, W., M.A., M.B., B.CHIR. (Cantab.), D.P.M. | Consultant Psychiatrist, Devon & Exeter Clinical Area. | Senior Psychiatric Registrar, St. Bartholomew's Hospital and the North Middlesex Hospital, London. |
| MCCORMICK, A. J. A., M.B., CH.B. (Edin.), F.R.C.S. (Edin.), D.O.M.S. | Ophthalmologist, Torquay (School Eye Service). | Senior Ophthalmic Registrar, United Bristol Hospitals. |
| HALL, G. H., M.B., B.S. (London), M.R.C.P. (London). | Senior Medical Registrar, Royal Devon & Exeter Hospital (joint appt. with the United Bristol Hospitals). | Medical Registrar, University College Hospital. |
| SIME, D. A., M.B., CH.B. (Edin.). | Senior Hospital Medical Officer Royal Western Counties Institution, Starcross. | Assistant Psychiatrist & Deputy Physician Supt. Aston Hall Mental Def. Hospital, Derbyshire. |
| HARRIS, R. H., M.B., B.S. (London), D.P.M. | Assistant Psychiatrist, Digby-Wonford Hospital, Exeter. | J.H.M.O. Moorhaven Hospital, Ivybridge, S. Devon. |
| ARTHUR, L. J. H., M.B., B.CHIR. (Cantab.), D.R.C.O.G., M.R.C.P. (London). | Clinical Asst. in Medicine, Tiverton District & Post Hill Hospitals, Tiverton. | In general practice in Tiverton. |
| JOHNS, E. R., M.B., B.S. (London), L.M.C.C., D.R.C.O.G. | Registrar in Obstetrics and Gynaecology, Redruth Hospital. | House Surgeon, Chase Farm Hospital, Enfield. |
| LEDWICH, J. R., M.B., CH.B. (Manchester). | Medical Registrar, Royal Cornwall Infirmary, Truro. | Resident Medical Officer, Oldham Royal Infirmary. |
| TANDON, P. N., M.B., B.S. (Lucknow). | E.N.T. Registrar, South Devon & East Cornwall Hospital, Plymouth. | S.H.O. in E.N.T. Surgery, Bristol General Hospital. |

